# Investigating the Degradation of EUV Transmittance of an EUV Pellicle Membrane

**DOI:** 10.3390/membranes13010005

**Published:** 2022-12-21

**Authors:** Seong Ju Wi, Yong Ju Jang, Dong Gi Lee, Seon Yong Kim, Jinho Ahn

**Affiliations:** 1 Division of Materials Science and Engineering, Hanyang University, Seoul 04763, Republic of Korea; 2 EUV-IUCC (Industry University Cooperation Center), Hanyang University, Seoul 04763, Republic of Korea; 3 Division of Nanoscale Semiconductor Engineering, Hanyang University, Seoul 04763, Republic of Korea

**Keywords:** EUV pellicle, EUV transmittance, oxidation, sputtering, microstructure, diffusion

## Abstract

The extreme ultraviolet (EUV) pellicle is a freestanding membrane that protects EUV masks from particle contamination during EUV exposure. Although a high EUV transmittance of the pellicle is required to minimize the loss of throughput, the degradation of EUV transmittance during the extended exposure of the pellicle has been recently reported. This may adversely affect the throughput of the lithography process. However, the cause of this phenomenon has not yet been clarified. Therefore, we investigated the cause of the degradation in the EUV transmittance by observing the compositional change when the Ru/SiN_x_ pellicle composite was heated in an emulated EUV scanner environment. The Ru thin film that was deposited at high pressure had more void networks but was not oxidized, whereas the SiN_x_ thin film was oxidized after heating. This was because the void network in the Ru thin film served as a preferential diffusion path for oxygen and caused oxidation of the SiN_x_ thin film. It was confirmed that the degradation of the EUV transmittance was due to the oxidation of SiN_x_. The results verified the effect of diffusivity in the thin film due to the void network on oxidation and EUV transmittance.

## 1. Introduction

At 7 nm technology nodes and beyond, the high-volume manufacturing (HVM) of semiconductor logic devices and dynamic random-access memory (DRAM) has been performed using extreme ultraviolet (EUV) lithography [1,2]. The EUV pellicle is an extremely thin film located at a stand-off distance from the EUV mask, which defocuses particles generated during the exposure process and prevents wafer defects [3]. The mechanical, thermal, and chemical durability of the pellicle inside an EUV scanner is essential. In addition, the EUV pellicle requires a transmittance higher than 90% at a 13.5 nm wavelength to minimize the loss of throughput resulting from the absorption of EUV photons by the pellicle [3,4,5,6,7]. However, the EUV transmittance gradually decreases as the exposure time for the EUV pellicle increases. This adversely affects the throughput of the exposure process [3,8].

Intrinsic and extrinsic defects inevitably exist in thin films deposited by non-equilibrium processes, such as sputtering, which is a physical vapor deposition technology used in various fields [9,10]. In the case of a void network formed in a thin film with a columnar structure, the diffusivity is much higher than that at the grain boundary [11]. Therefore, the void network may act as a preferred diffusion path for oxygen to cause oxidation. During the exposure process, this phenomenon is expected to cause the degradation of the EUV transmittance of the EUV pellicle.

Herein, the pellicle was heated to a high temperature in an environment similar to that inside an EUV scanner. The composition and EUV transmittance before and after heating were analyzed. In addition, the effect of the diffusion process in ruthenium (Ru) thin films on the oxidation of the pellicle was assessed by comparing the compositional changes to its microstructure. According to past research, it is argued that the degradation of EUV transmittance can be prevented by forming a denser passivation layer.

## 2. Materials and Methods

Ru thin films with a thickness of a few nanometers and high emissivity were used as thermal emission layers for EUV pellicles. In addition, silicon nitride (SiN_x_) thin films were utilized as a base material for EUV pellicles because of their high EUV transmittance and mechanical durability compared with other materials [12,13,14]. Therefore, measurements and evaluation of Ru/SiN_x_ pellicle composites based on Ru and SiN_x_ thin films were performed in this study.

To fabricate the Ru/SiN_x_ pellicle composite, low-pressure chemical vapor deposition (LPCVD) was performed using dichlorosilane (SiH_2_Cl_2_) and ammonia (NH_3_) at 800 °C. A 40 nm thick silicon-rich silicon nitride (SiN_x_, x~1.0) film was deposited onto a (100) p-type silicon wafer. The photoresist was coated on the back side to obtain a membrane with an area of 10 × 10 mm^2^. Thereafter, the exposure and development processes were performed. The membrane area was patterned by a reactive ion etching (RIE) process using CF_4_, CHF_3_, and O_2_ as the reactant gas and Ar as the carrier gas. Subsequently, a SiN_x_ free-standing membrane was fabricated by wet etching a Si wafer at 60 °C using a 30 wt.% KOH solution. The SiN_x_ membrane was etched to a thickness of 34 nm by the KOH solution. A 3 nm thick Ru thin film was deposited on the SiN_x_ membrane via DC magnetron sputtering. The sputtering target, of diameter 4 inches, was of high purity (99.95%). The Ru thin film was prepared at working pressures varying from 0.5 × 10^−3^ to 2 × 10^−3^ Torr. The chamber was evacuated to a base pressure of less than 2 × 10^−7^ Torr. The thickness of the Ru/SiN_x_ film as a function of the deposition conditions was confirmed by cross-view images obtained using transmission electron microscopy (TEM; JEOL, Tokyo, Japan). In addition, plan-view TEM images of the Ru thin films deposited on SiN_x_ grids (TED PELLA, Altadena, CA, USA) were obtained to analyze the void network.

**Figure 1:**
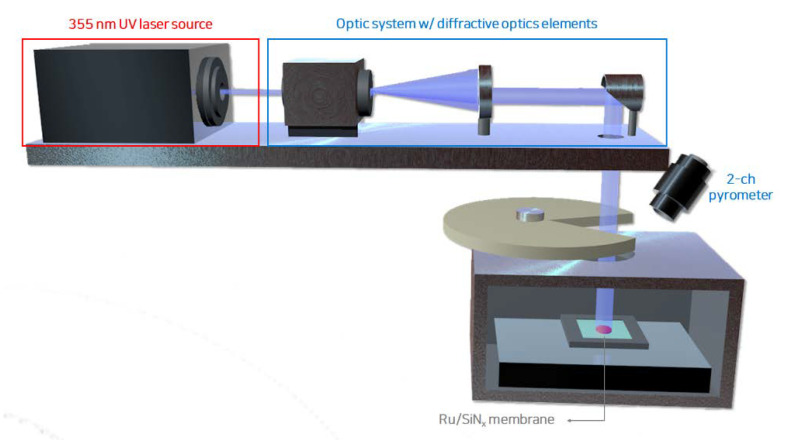
Schematic of the laser-pulsed heating equipment with a high-vacuum environment.

A high-vacuum environment was maintained inside the EUV scanner to minimize the atmospheric absorption of EUV light, and the pellicle was periodically exposed to EUV light with a uniform profile during the exposure process. Figure 1 presents a schematic of the heat load tester with a 355 nm laser used to heat the pellicle composite in an environment similar to the EUV exposure process. A diffractive optics element was used such that the laser was uniformly incident on the pellicle. The pellicle was periodically exposed by a rotating slit in a high-vacuum chamber of 6 × 10^−6^ Torr. The heated temperature was measured using a 2-channel pyrometer. The thermal load absorbed by the pellicle composite was calculated using the laser power, beam size, and absorbance at a wavelength of 355 nm [7]. In this study, the pellicle composite was heated for 3, 6, and 9 h under an absorbed thermal load of 0.8 W/cm^2^ with an average peak temperature of 450 °C.

The composition of the thin film before and after laser-pulsed heating was analyzed using X-ray photoelectron spectroscopy (XPS; Thermo Fisher Scientific Co., Waltham, MA, USA) with an Al Kα X-ray source (1486.6 eV). The survey spectra were acquired at a pass energy of 30 eV using an analysis area of 200 μm. The obtained spectra were calibrated based on the Au 4f peak.

The EUV transmittance (EUVT) of the pellicle composite as a function of the pulsed heating time was measured using coherent scattering microscopy (CSM) with an EUV source. The EUVT was calculated by comparing the number of photons reflected from an EUV mirror composed of 40 pairs of Mo/Si multilayers with and without pellicles [15,16]. The EUV transmittance was determined from the average value by repeating the measurements 10 times at each of the three points in the membrane area to obtain reliable results.

## 3. Results and Discussion

According to the structure zone model (SZM), the microstructure of the thin film deposited by sputtering is determined by the adatom energy and diffusion of the adatom absorbed on the substrate, which varies depending on the deposition conditions [9,10,17]. The Zone 1 structure is formed at a high working pressure, low sputtering power, and low substrate temperature with low adatom energy and consists of irregular columnar structures with a void network. In this study, we expected the formation of the Zone 1 structure as the Ru thin film was being deposited at room temperature.

**Figure 2:**
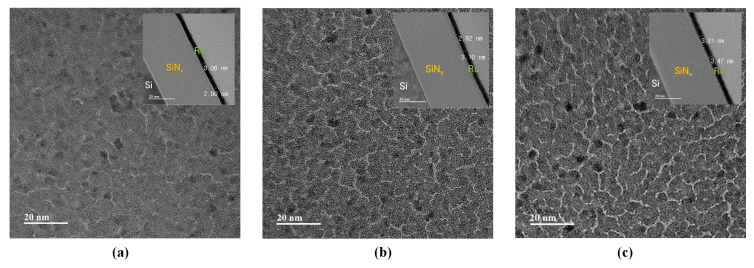
TEM analysis results of the Ru thin film deposited by sputtering with a working pressure of (**a**) 0.5, (**b**) 1.0, (**c**) 2.0 mTorr.

Figure 2 shows the top-view and cross-sectional TEM images of the Ru/SiN_x_-composite membranes deposited at working pressures of 0.5, 1.0, and 2.0 mTorr, respectively. Each figure showed the thickness and void network of the Ru thin film at respective sputtering conditions. The area of the white region represents the void network in the thin film. As the working pressure increased, the void network in the thin film gradually increased along with the intensifying shadowing effect. A void network with low activation energy for oxygen diffusion can serve as a preferential diffusion path for oxygen at high temperatures. Therefore, oxygen diffusion at the Ru/SiNx interface at high temperatures is expected to be more active as the thin film is deposited under high-working-pressure conditions.

**Figure 3:**
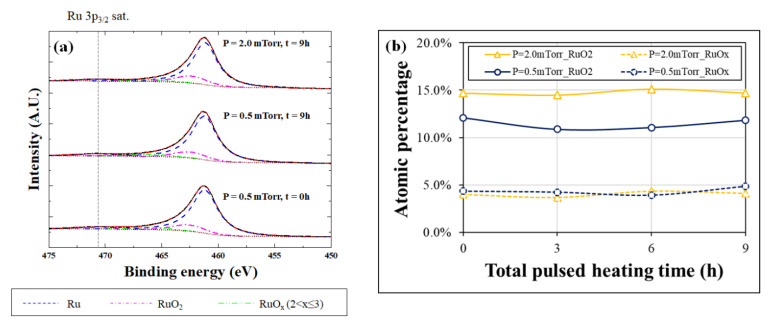
(**a**) Ru 3p_3/2_ spectra before and after laser pulsed heating for 9 h according to the working pressure. (**b**) Atomic percentage of Ru oxide species for Ru thin film deposited at working pressures of 0.5 mTorr and 2.0 mTorr as a function of laser-pulsed heating time.

The oxidation of the SiN_x_ thin film can be analyzed from the Si 2p and O 1s spectra because the Ru thin film is relatively thin (3 nm). Therefore, the oxidation characteristics of the bilayer membrane in a high-vacuum and high-temperature environment depending on Ru deposition conditions were investigated using Ru 3p, Si 2p, and O 1s spectra. Figure 3a shows the Ru 3p_3/2_ spectra before and after laser-pulsed heating for the various working pressures. The possible oxidation states of Ru were RuO_2_ and RuO_x_ (2 < x ≤ 3). The Ru, RuO_2_, and RuO_x_ peaks were marked at binding energies of 461.2, 462.5, and 465.9 eV, respectively [18,19,20]. In addition, the area ratio of Ru 3p_1/2_ was constrained to 1:2 considering spin–orbit splitting [18,21,22]. Figure 3b shows the atomic percentages of the Ru oxide species according to the total laser-pulsed heating time. After pulsed heating, the fractions of RuO_2_ and RuO_x_ were maintained at an average of 12.1% and 4.9%, respectively, for the thin film deposited at a working pressure of 0.5 mTorr. Additionally, the fractions of RuO_2_ and RuO_x_ were at an average of 15.1% and 4.3% at 2.0 mTorr, respectively. The atomic percentage of the Ru oxide species was almost constant, even after prolonged heating. The Ru oxide species were estimated to be a native oxide that was inevitably formed regardless of the heating time.

The oxidation of the SiN_x_ thin film located under Ru can be analyzed because the average depth of analysis for XPS measurement is approximately 5 nm. In the case of non-stoichiometric amorphous SiN_x_, Si and N atoms are bonded to form any one of the five tetrahedra of Si-Si_4−x_N_x_ (where x = 0, 1, …, 4) using a random-bonding model [23,24,25,26]. There are many Si-Si bonds in the Si-rich SiN_x_ thin film, which can lead to oxidation caused by diffused oxygen in the Ru/SiN_x_ interface and the formation of a Si-SiN_x_O_4−x_ (where x = 0, 1, …, 4) tetrahedron.

**Figure 4:**
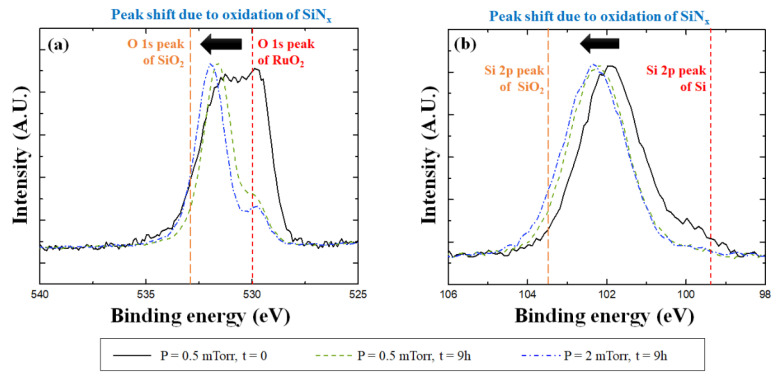
(**a**) O 1s and (**b**) Si 2p XPS spectra depending on working pressure before and after laser-pulsed heating with a peak temperature of 450 °C for 9 h.

Figure 4a presents the O 1s spectra of the thin film heated for 9 h depending on the Ar working pressure. In the case of the thin film deposited at a 0.5 mTorr working pressure, the peak position shifted to 531.6 eV as the peak intensity of oxidized SiN_x_ increased after laser-pulsed heating. The peak position of the thin film deposited at a 2.0 mTorr working pressure was shifted to 532.0 eV after heating. This was expected since the Si-SiN_x_O_4−x_ bonding state was formed as the Si-rich SiN_x_ was further oxidized. Figure 4b shows the Si 2p spectra, which shows a skewed peak shape between 98.8 eV and 104.0 eV for the reference sample. This implies the possibility of Si-rich SiN_x_ with a tetrahedral structure considering the peak positions of Si and SiO_2_ [24]. The binding energy increased as the Si-O bonds were formed in the SiN_x_ thin film after pulsed heating. In particular, the peak of the thin film deposited at 2.0 mTorr shifted more than the peak of the thin film deposited at 0.5 mTorr. This was due to more Si-O bonds formed in the thin film deposited at a 2.0 mTorr working pressure.

**Figure 5:**
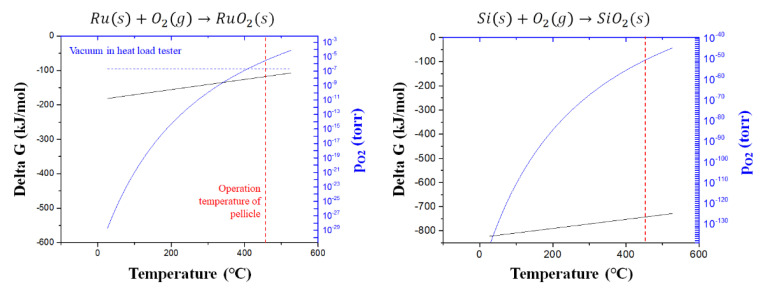
Gibbs free energy and equilibrium oxygen partial pressure based on the oxidation reaction temperature of Ru and Si.

The oxidation of the Ru and SiN_x_ thin films after laser-pulsed heating was investigated using a thermodynamic approach. Equilibrium oxygen partial pressures can be calculated using Equation (1) based on the Gibbs free energy of the stoichiometric oxide formation [27].

(1) $$\Delta G{}^{\circ}=RT\ln p_{O2\left(eq.T\right)}\;\mathrm{for}\;Ru\;\left(s\right)+O_{2}\;\left(g\right)\to RuO_{2}\;\left(s\right)$$

where R is the ideal gas constant, T is the temperature of the reaction system, and $p_{O2\left(eq.T\right)}$ is the equilibrium oxygen partial pressure at T. Equation (1) can be applied to this experimental condition because the change in volume of the condensed phase is negligible. Figure 5 shows the Gibbs free energy for the oxidation reaction of Ru and Si according to the temperature and calculated equilibrium oxygen partial pressure. The equilibrium temperature of the Ru oxidation reaction was calculated to be 409 °C, assuming the oxygen fraction in the high-vacuum environment is the same as that in the atmosphere. Above 409 °C, Ru oxide is reduced [20,28]. In this study, Ru oxide was reduced when the pellicle composite was heated to 450 °C and the Ru thin film was oxidized again at room temperature after laser-pulsed heating. Therefore, even when the pellicle composite was heated for a long time, the atomic percentage of the Ru oxide species remained constant. In the case of Si-rich SiN_x_ thin films with Si-Si bonding states, the Si bonds with relatively low bonding energies were broken and Si-O bonds were formed [25]. The equilibrium oxygen partial pressure of the reaction for Si oxidation was calculated to be approximately 10–50 Torr at 450 °C, which is much lower than the oxygen partial pressure in a high-vacuum environment. Therefore, the Si-rich SiN_x_ thin film was oxidized during laser-pulsed heating.

As shown in Figure 2, larger void networks were formed in the Ru thin film as the Ar working pressure increased. It is expected that the diffused oxygen caused the oxidation of the SiN_x_ thin film as the void network acted as a preferential diffusion path for oxygen. The oxygen diffusivity in the Ru thin film according to temperature is expressed by Equation (2) of an Arrhenius form.

(2) $$D=D_{0}e^{-\frac{E_{a}}{kT}}$$

where $D_{0}$ is the pre-exponential factor, $E_{a}$ is the activation energy, k is the Boltzmann constant, and T is the temperature of the reaction system. According to Equation (2), the oxygen diffusivities of the ideal Ru thin film were 1.9 × 10^−25^ and 1.6 × 10^−14^ m^2^/s at 25 °C and 450 °C, respectively. Therefore, oxygen can diffuse into the Ru thin film, even when considering the difference in oxygen partial pressure between the atmosphere and high vacuum [29]. The results suggest that the Si-rich SiN_x_ thin film should be further oxidized because the effective diffusivity increases owing to the void network formation as the pressure increases.

**Figure 6:**
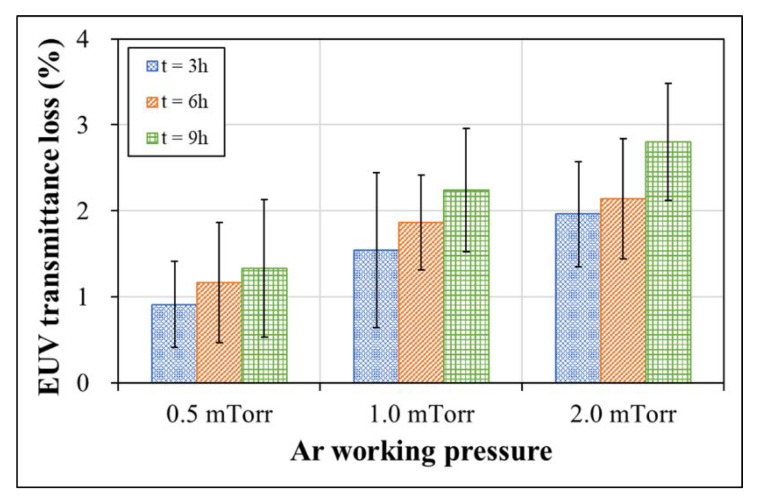
EUV transmittance loss of Ru/SiN_x_ pellicle composite deposited by various working pressures as a function of total laser-pulsed heating time.

The oxidation of the EUV pellicle generally adversely affects EUV transmittance, as most oxides have a high EUV absorbance. For example, when SiO_2_ and Si_2_N_2_O are formed by the oxidation of Si and Si_3_N_4_, the EUV transmittance decreases by 0.9% and 0.4% per 1 nm thickness, respectively [30]. Because Si-rich SiN_x_ was used in this study, the EUV transmittance may be significantly lower than that of stoichiometric Si_3_N_4_. Figure 6 shows the EUV transmittance loss of the pellicle composite as a function of the working pressure and total laser-pulsed heating time. Before laser-pulsed heating, the measured EUV transmittances of the pellicle composite deposited at working pressures of 0.5, 1.0, and 2.0 mTorr were 80.5, 80.1, and 79.1%, respectively. EUV transmittance losses of 1.3, 2.2, and 2.8% were observed as the SiN_x_ thin film was oxidized after pulsed heating for 9 h.

Oxidation of the EUV pellicle during exposure process can have a fatal effect on the EUV transmittance performance. However, the thickness of the thin film is not a controllable parameter because the EUV pellicle must have a high EUV transmittance to minimize the loss of throughput. Therefore, forming a dense passivation layer with a minimal void network is imperative to prevent the oxidation of EUV pellicle.

## 4. Conclusions

In this study, the chemical stability and loss of EUV transmittance of the Ru/SiN_x_ pellicle composite were investigated. A Ru/SiN_x_ bilayer membrane was fabricated, and the void network of the Ru thin film was confirmed according to the deposition conditions. Laser-pulsed heating was performed in an environment similar to the inside of the EUV scanner to analyze the change in the bonding state and EUV transmittance due to oxidation of the pellicle composite. The Ru thin film was not further oxidized by laser-pulsed heating because the heating temperature was in the thermodynamic reduction region. In contrast, Si-SiN_x_O_4−x_ was formed by the oxidation of Si-rich SiN_x_ after laser-pulsed heating. The film also tended to be oxidized further as the working pressure increased. This was due to the formation of more void networks, which served as preferential diffusion paths for oxygen. The EUV transmittance of the Ru/SiN_x_ pellicle composite degraded owing to the oxidation of the Si-rich SiN_x_ thin film after laser-pulsed heating. Therefore, it was expected that the degradation of EUV transmittance during the EUV exposure process was due to the oxidation of the EUV pellicle. Therefore, to prevent oxidation of the EUV pellicle and minimize the formation of a void network, the deposition conditions must be optimized, such as using high sputtering power, low working pressure, and higher substrate-heating temperatures to form a dense passivation layer.
